# Knowledge, attitude, practice and associated factors of oxygen therapy among health professionals in Ethiopia: A systematic review and meta-analysis

**DOI:** 10.1371/journal.pone.0309823

**Published:** 2024-09-06

**Authors:** Zewdu Bishaw Aynalem, Mekides Nigusu Abera, Birhaneslasie Gebeyehu Yazew, Melsew Dagne Abate, Ayalew Kassie, Fentahun Meseret, Ahmed Nuru Muhamed, Gebremeskel Kibret Abebe, Meseret Mekuriaw Beyene, Tilahun Degu Tsega

**Affiliations:** 1 Department of Nursing, College of Medicine and Health Sciences, Injibara University, Injibara, Ethiopia; 2 Department of Nursing, Bahir Dar Health Science College, Bahir Dar, Ethiopia; 3 Department of Pediatrics and Child Health Nursing, School of Nursing and Midwifery, College of Health and Medical Science, Haramaya University, Harar, Ethiopia; 4 Department of Nursing, College of Medicine and Health Science, Wolkite University, Wolkite, Ethiopia; 5 Department of Emergency and Critical Care Nursing, School of Nursing, College of Health Sciences, Woldia University, Woldia, Ethiopia; 6 Department of Public Health, College of Medicine and Health Sciences, Injibara University, Injibara, Ethiopia; Ministry of Health, General Health Directorate of Raparin and University of Raparin, IRAQ

## Abstract

**Background:**

Oxygen therapy is a life-saving intervention used in various healthcare settings to maintain adequate tissue oxygenation while minimizing cardiopulmonary work. Its effective and safe administration depends largely on the knowledge, attitudes, and practices of health professionals. However, there are no pooled studies that examined these skills in the context of Ethiopia. Therefore, this study aimed to assess the pooled prevalence of health professionals’ knowledge, attitude, practice, and determinant factors about oxygen therapy in Ethiopia.

**Methods:**

The databases PubMed, Web of Science, Scopus, Hinari, Science Direct, African Journal of Online, and Google Scholar were used to search for published studies; Direct Google searches and institutional repositories were used to search for unpublished studies. Duplicate studies were eliminated with Endnote X8 and reported according to PRISMA guidelines. The quality of the included studies was assessed using the Joanna Briggs Institute critical appraisal checklist. A random-effects model was used to estimate the pooled prevalence of KAP among health professionals. Heterogeneity was assessed using Cochran’s Q test and I^2^ statistics. Publication bias was checked by visual inspection of a funnel plot and Egger’s regression test. STATA version 11 software was used for statistical analysis.

**Results:**

A total of 14 studies with 2,960 participants for knowledge and practice and 9 studies with 1,991 participants for attitude were used to estimate the pooled prevalence of KAP among health professionals. The pooled prevalence of good knowledge, positive attitude, and good practice regarding oxygen therapy were 52.13% (95% CI: 43.88, 60.39), 55.08% (95% CI: 50.80, 59.35%), and 48.94% (95% CI: 41.14, 56.74) respectively. Both good knowledge and positive attitude were affected by the availability of oxygen therapy guidelines, with adjusted odds ratios (AOR) of 6.11 (95% CI: 2.45, 15.22) and 2.17 (95% CI: 1.39, 3.39) respectively. Additionally, good knowledge (AOR: 4.31, 95% CI: 1.53, 12.11), training (AOR: 4.09, 95% CI: 2.04–8.20), and having an adequate oxygen supply and delivery system (AOR: 3.12, 95% CI: 1.92–5.07) were statistically associated with good practice.

**Conclusion and recommendations:**

The national pooled prevalence of good knowledge, positive attitude, and good practice among health professionals was low. Therefore, thorough monitoring, supervision, and evaluation of their oxygen therapy is highly recommended for all stakeholders. Yet again, we strongly advise that the identified factors be improved by organizing training sessions, making oxygen therapy guidelines available, and maintaining an adequate oxygen supply system.

**Trial registration:**

The review protocol was registered in the international prospective register of systematic reviews with registration number PROSPERO: CRD42023486036.

## Introduction

In many healthcare settings, oxygen therapy is a life-saving intervention that helps minimize cardiopulmonary work while maintaining proper tissue oxygenation [1]. According to the WHO, it is a must-have drug that needs to be administered at concentrations greater than those found in the ambient air [2]. Similar to other medications, oxygen therapy should only be started when there is an obvious indication such as myocardial infarction and stroke [3], high blood pressure, metabolic acidosis [4, 5], acute respiratory distress syndrome [6], and chronic obstructive pulmonary disease [7, 8]. Oxygen is therefore given to prevent hypoxia and its consequent complications [9].

Because of its beneficial and harmful biological effects, oxygen is widely recognized as a double-edged sword [10]. Therefore, physicians need to prescribe oxygen with precise instructions regarding dosage, duration of use, appropriate delivery device, and the intended oxygen saturation range [11, 12]. To guarantee correct administration, it should be monitored with a pulse oximeter [13, 14]. But, if administered improperly this drug may potentially inflict considerable harm [15, 16]. Indeed, giving too much oxygen promotes the production of superoxide and free radicals, which can lead to atelectasis, cardiac arrest, pulmonary vasoconstriction, lung damage, cell apoptosis, and necrosis [17–19]. Conversely, under-oxygenation can also cause disorientation, headaches, restlessness, tachycardia, tachypnea, dyspnea, changes in speech, visual disturbance, renal dysfunction, impaired mentation, and nausea and vomiting [20, 21]. As a result, it is imperative to recognize the negative effects arising from both under and over-oxygenation [15, 22].

The effective and safe administration of oxygen relies heavily on the knowledge, attitudes, and practices of health professionals [23–26]. Lack of these skills among healthcare professionals (HPs) can result in errors and incorrect administration of oxygen therapy. Such improper administration may potentially result in permanent retinal damage (retinopathy), leading to blindness [14, 27], and heightened morbidity and mortality rates [28]. Along with increasing admission rates to high-dependency units, it can also prolong hospital stays and surge the risk of death [29]. Moreover, it diminishes surfactant production and induces oxygen toxicity, both of which can cause the alveoli to collapse [30, 31].

Evidence showed that HPs have glaring gaps in their knowledge, attitudes, and practices regarding oxygen therapy, with variations observed across different countries [32–35]. In Tanzania, 37.5% of HPs had poor practices, and nearly half (46.2%) possessed poor knowledge [28]. In Rwanda, 37.64% of HPs lacked knowledge about oxygen therapy, 14.4% had negative attitudes, and 32.59% had poor practice [31]. In Pakistan, 75.6% of health professionals had good knowledge [36]. In Egypt, only a small percentage of health professionals, 10% and 5% had adequate practice and satisfactory knowledge, respectively [37]. Good oxygen knowledge, positive attitude, and good practice regarding oxygen therapy were found in 43.3%, 63.3%, and 45% of Eritrea’s HPs respectively [38]. In Ethiopia only 17.7% of HPs had good knowledge, 40.4% had good attitude, and 19.1% had good practice [24]. However, another study conducted in Ethiopia reported higher percentages, with around 55% of HPs having good knowledge, 54.6% exhibiting good attitudes, and 65.1% demonstrating good practices about oxygen therapy [39].

Although several individual studies have reported the prevalence of knowledge, attitude, and practice among health professionals in Ethiopia, no national study has shown the country’s KAP status concerning oxygen therapy. Moreover, oxygen is a valuable resource, especially in environments with limited resources like Ethiopia. This limited resource may be utilized needlessly and wastefully as a result of inadequate knowledge, a negative attitude, and inappropriate practices. By identifying gaps, oxygen can be used effectively and efficiently for the patients who truly need it, thereby preventing unnecessary wastage. Furthermore, assessing oxygen therapy knowledge, attitudes and practice provides insight into their continuous professional development (CPD) needs. By identifying areas where additional education or training is required, stakeholders can design intervention programs that meet those CPD needs. As a result, this lifelong learning and professional development can assist HPs in staying up-to-date and guaranteeing their proper oxygen administration. Thus, to fill the abovementioned gaps, this systematic review and meta-analysis was conducted to assess the pooled prevalence of knowledge, attitude, practice, and determinant factors of oxygen therapy among health professionals in Ethiopia.

## Materials and methods

### Reporting

To ensure transparency in the process, this protocol has been appropriately registered in the International Prospective Register of Systematic Reviews database (CRD42023486036). The reviewers also checked the DARE database (http://www.library.UCSF.edu) and the Cochrane Library to ensure this had not been done before and to avoid duplication. These checks confirmed that no similar studies had been conducted previously.

### Study selection and search strategy

This systematic review and meta-analysis were conducted per the Preferred Reporting Items for Systematic Reviews and Meta-Analyses (PRISMA) checklists [40]. All relevant and published articles in the following databases; PubMed, Web of Science, Scopus, Science Direct, Google Scholar, HINARI, and African Journal of Online were searched using a combination of Boolean operators (OR and AND) and keywords. The first search was conducted from November 23 to 29, 2023, and then updated from May 13 to 19, 2024. We reviewed gray literature via direct Google search. Research repositories of Ethiopian universities were also searched to identify unpublished studies. Bibliography lists of eligible studies were also reviewed to maximize the inclusion of relevant studies. The detailed search strategies are found in the Supporting information (S1 Table).

### Eligibility criteria

For inclusion of the literature, the following criteria were applied to the retrieved studies (i) being observational studies conducted in Ethiopia up to May 2024, (ii) being published in peer-reviewed journals and unpublished articles in English, (iii) being conducted among Ethiopian HPs (iv) being concerned with any of the KAP of oxygen therapy and (v) having full-text access. In addition, (i) articles published in languages other than English (ii) studies except for observational studies, such as reviews, case series, editorial, commentary, qualitative studies, studies with missing outcomes of interest, and articles that failed to fulfill the aforementioned eligibility criteria were eliminated from this review. An attempt was made to contact the corresponding authors using the email address or phone number provided in the published articles.

### Study selection and data extraction

All the retrieved studies were exported and duplicates were removed by using the EndNote X8 citation manager. Then, four authors (ZB, AK, MM, and MN) independently conducted a preliminary selection by reading the titles and abstracts of the identified articles using the eligibility criteria. Subsequently, four authors (AN, FM, GK, and MD) thoroughly reviewed the full text of the articles for further screening concerning their objectives, methods, population, and key findings. Any discrepancies were resolved through discussions and consultations with the remaining two investigators (TD and BG). A predefined Microsoft Excel 2016 format was used to extract the data from selected studies under the following headings: author name, year of publication, study setting, study design, data collection method, study population, sample size, and prevalence of good knowledge, positive attitude, and good practice.

### Outcome measurements

This systematic review and meta-analysis had two main outcomes. The primary outcomes were the pooled prevalence knowledge, attitude, and practice among health professionals in Ethiopia, whereas, factors associated with KAP of oxygen therapy among Ethiopian health professionals, which were assessed using the odds ratio were another outcome. Health professionals’ knowledge, attitude, and practice about oxygen therapy were measured as follows:

#### Knowledge

Knowledge was assessed based on questions about oxygen administration that included considerations of oxygen as a drug, the definition, goals, purposes, indication and contra-indication of oxygen therapy, targeted oxygen saturation and partial pressure ranges, and familiarity with oxygen delivery devices. Knowledge was defined as good if the respondents scored above the mean score.

#### Attitude

Attitude was assessed by using questions about oxygen administration order, the importance of continuous oxygen administration over intermittent therapy and safety precautions when administering oxygen, attitude towards compliance to oxygen therapy protocols, sense of responsibility to document observations during oxygen administration, confidence and skill to deal and prevent under or over oxygenation. A respondent whose score was above the mean level was classified as having a positive attitude.

#### Practice

Practice was assessed using questions on assembling necessary equipment before oxygen administration, selecting the right oxygen delivery devices for patient needs, care during oxygen therapy, oxygen therapy monitoring techniques, and timing of weaning and cessation of oxygen therapy. The respondents were categorized as having good practice if he/she scored above the mean.

### Quality assessment

**Fig 1:**
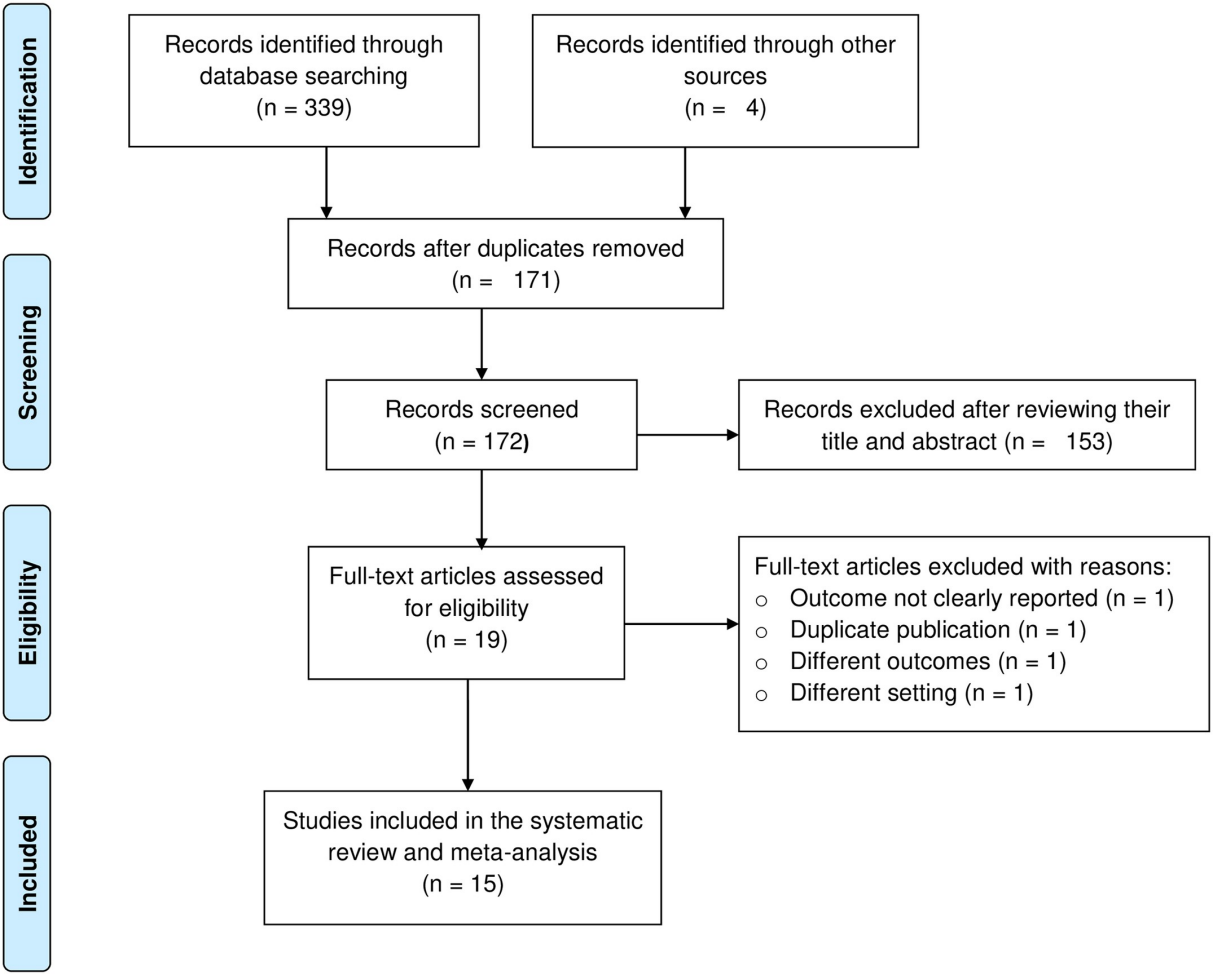
PRISMA flow diagram of study selection, Ethiopia, 2023.

The Joanna Briggs Institute quality appraisal checklist was used [41, 42] (S2 Table). When there was disagreement, all authors discussed it and came to a resolution. There were nine parameters on the critical appraisal checklist with options of “yes, no, unclear, and not applicable”. The following questions were included in the quality parameters: (1) was the sample frame appropriate to address the target population?; (2) Were study participants sampled appropriately?; (3) Was the sample size adequate?; (4) Were the study subjects and the setting described in detail?; (5) Was the data analysis conducted with sufficient coverage of the identified sample?; (6) Were the valid methods used for the identification of the condition?; (7) Was the condition measured in a standard, reliable way for all participants?; (8) Was there appropriate statistical analysis?; and (9) Was the response rate adequate, and if not, was the low response rate managed appropriately? The quality of each included article was classified as higher (>80%), moderate (65%-80%), or low (< 60%). Studies with a score greater than or equal to 60% were included. The overall study selection process was presented using the PRISMA statement flow diagram (Fig 1).

### Heterogeneity and publication bias

Cochran’s Q statistics was used to assess statistical heterogeneity between studies, and the heterogeneity was quantified by the I^2^ statistic value, where 25%, 50%, and 75% represent low, medium, and high heterogeneity, respectively [43]. A p-value less than 0.05 was used to declare heterogeneity. A random effect model was used to reduce the heterogeneity of studies [44]. Funnel plots and Egger’s regression test were used to check for publication bias, with p <0.05 considered to exist potential publication bias [45, 46]. Moreover, subgroup, meta-regression, and sensitivity analysis were carried out.

### Data analyses

A Microsoft Excel spreadsheet was utilized to extract the data, which was then imported into STATA version 11 for analysis. Tables, figures, and forest plots were used to describe the results of the study. A random-effects model was used to estimate the pooled prevalence of good knowledge, positive attitude, and good practice among HPs as this is the recommended method in a meta-analysis to account for the observed variability between studies [47]. Statistical heterogeneity was assessed by Cochran’s Q statistics and the I^2^ test. The sub-group analysis was also performed based on the region of the study conducted, study population, sample size and study quality. A funnel plot was constructed to visually examine the publication bias. In addition, Egger’s regression test was used to detect evidence of publication bias. A p-value of < 0.05 was considered to indicate the presence of significant publication bias. A pooled odds ratio with a 95% confidence interval was used to quantify the level of association for factors affecting the knowledge, attitude, and practice of oxygen therapy among HPs. Finally, a p-value of less than 0.05 at 95% CI was used to declare statistical significance.

## Results

### Selection and identification of studies

A total of 343 articles were identified in major electronic databases and other relevant sources. One hundred and seventy-one articles were eliminated due to duplication and 172 studies were retained for further review. One hundred fifty-three of them were excluded because their titles and abstracts didn’t fit the criteria. Four of the remaining 19 were removed due to inconsistencies in settings, study population, and outcome of interest (S3 Table). In the end, 15 articles were chosen and included in the final review. Generally, full screening was done based on the PRISMA flow diagram (Fig 1).

### Characteristics of included studies

A total of 15 articles with 3,360 participants were included in this systematic review and meta-analysis. Females accounted for 1,759 (52.35%) of the study participants. The mean age of the study participants was 30.28 (SD ±3.82) years old. Only 556 participants (16.55%) had diplomas, while the majority of them, 2,806 (83.45%), held BSc or higher degrees. Regarding work experience, 1,617 (48.13%) of them had less than 5 years, 1,241 (36.93%) had 5–10 years, and the remaining 502 (14.94%) had over 10 years. All included studies were cross-sectional and the sample size ranged from 102 [48] and 422 [32]. About the regional distribution of the included articles, four were studied in Addis Ababa [24, 30, 49, 50], three in the Southern Nations, Nationalities and Peoples Region (SNNPR) [48, 51, 52], and one each in the regions of Harari [32] and Tigray [53]. An additional six were studied in the Amhara region [35, 39, 54–57] (Table 1).

**Table 1:** Characteristics of the studies included in the systematic review and meta-analysis.

No	Author	Year	Region	Study design	Sample size	Quality score	Focused Group	Prevalence of		
								Good K	Favorable A	Good P
1.	Argaw et al. [24]	2023	Addis Ababa	CS	141	8	Doctors	17.7	40.4	19.1
2.	Lemma G [30]	2015	Addis Ababa	CS	152	7	Nurses and Midwives	36.2	53.3	43.4
3.	Jamie A [32]	2021	Harari	CS	422	8	Nurses	61.4	NR	47.5
4.	Zeleke & Kefale [35]	2021	Amhara	CS	105	8	Nurses	52	NR	33
5.	Demilew et al. [39]	2022	Amhara	CS	218	9	Physicians, Anesthetists, Nurses, Midwives, IESO	54.6	54.6	65.1
6.	Mezgebe T [48]	2022	SNNPR	CS	102	7	Nurses and doctors	52.9	NR	56.9
7.	Argeta et al. [51]	2022	SNNPR	CS	268	7	Nurses	55.6	60.8	73.9
8.	Kahsay et al. [53]	2021	Tigray	CS	180	8	Nurses	58.9	67.8	56.8
9.	Abitew K [54]	2022	Amhara	CS	244	8	Nurses	55.3	56.1	62.7
10.	Bizuneh et al. [55]	2022	Amhara	CS	400	9	Nurses	NR	NR	47
11.	Getahun et al. [56]	2022	Amhara	CS	400	9	Nurses	33	53.8	NR
12.	Getnet A [57]	2023	Amhara	CS	190	8	Nurses	76.8	55.8	36.8
13.	Kassaw et al. [49]	2024	Addis Ababa	CS	198	8	Nurses and Midwives	59.1	51.5	36.9
14.	Dansa A et al. [50]	2024	Addis Ababa	CS	166	8	Nurses	60.8	NR	54.2
15.	Bezawit B [52]	2023	SNNPR	CS	174	8	Nurses	55.7	NR	55.1

**Abbreviations:** A, attitude; CS, cross-sectional; IESO, integrated emergency surgical officers; K, knowledge; NR, not reported; P, practice; SNNPR, Southern Nation, Nationalities, and Peoples’ Region.

### Health professionals’ knowledge, attitude and practice toward oxygen therapy

**Fig 2:**
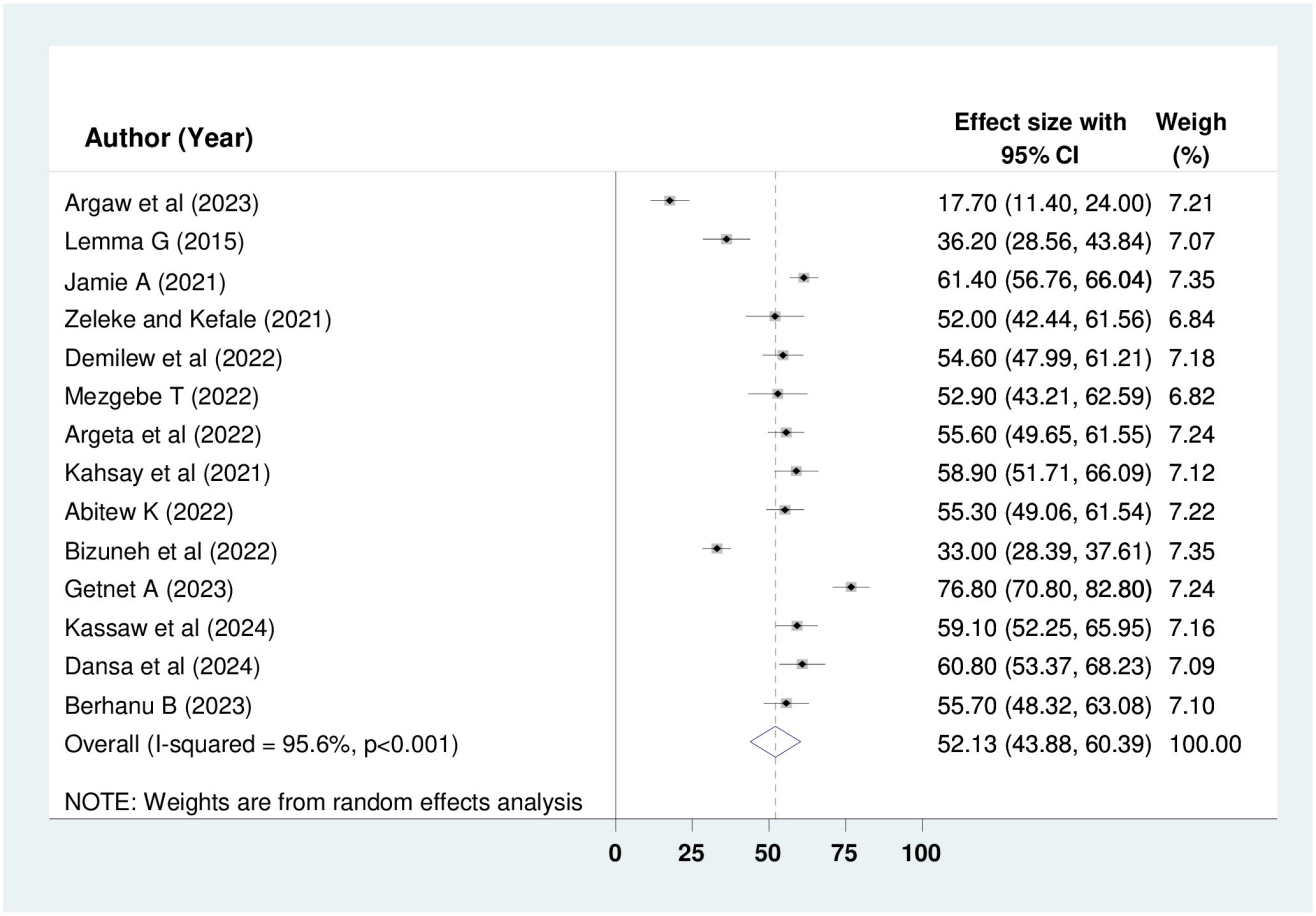
Forest plot showing the pooled prevalence of knowledge about oxygen therapy among HPs in Ethiopia.

**Fig 3:**
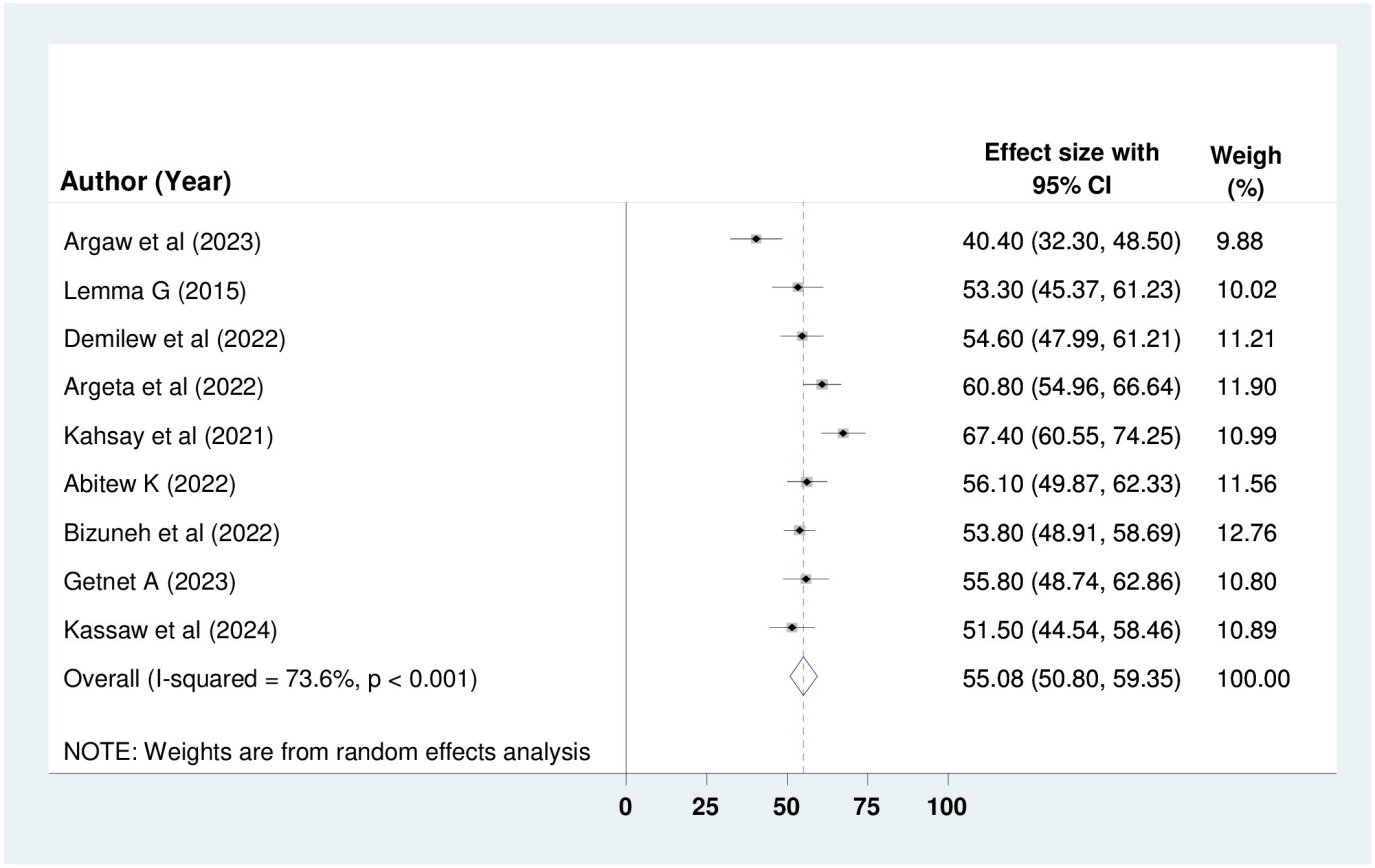
Forest plot showing the pooled prevalence of attitude towards oxygen therapy among HPs in Ethiopia.

**Fig 4:**
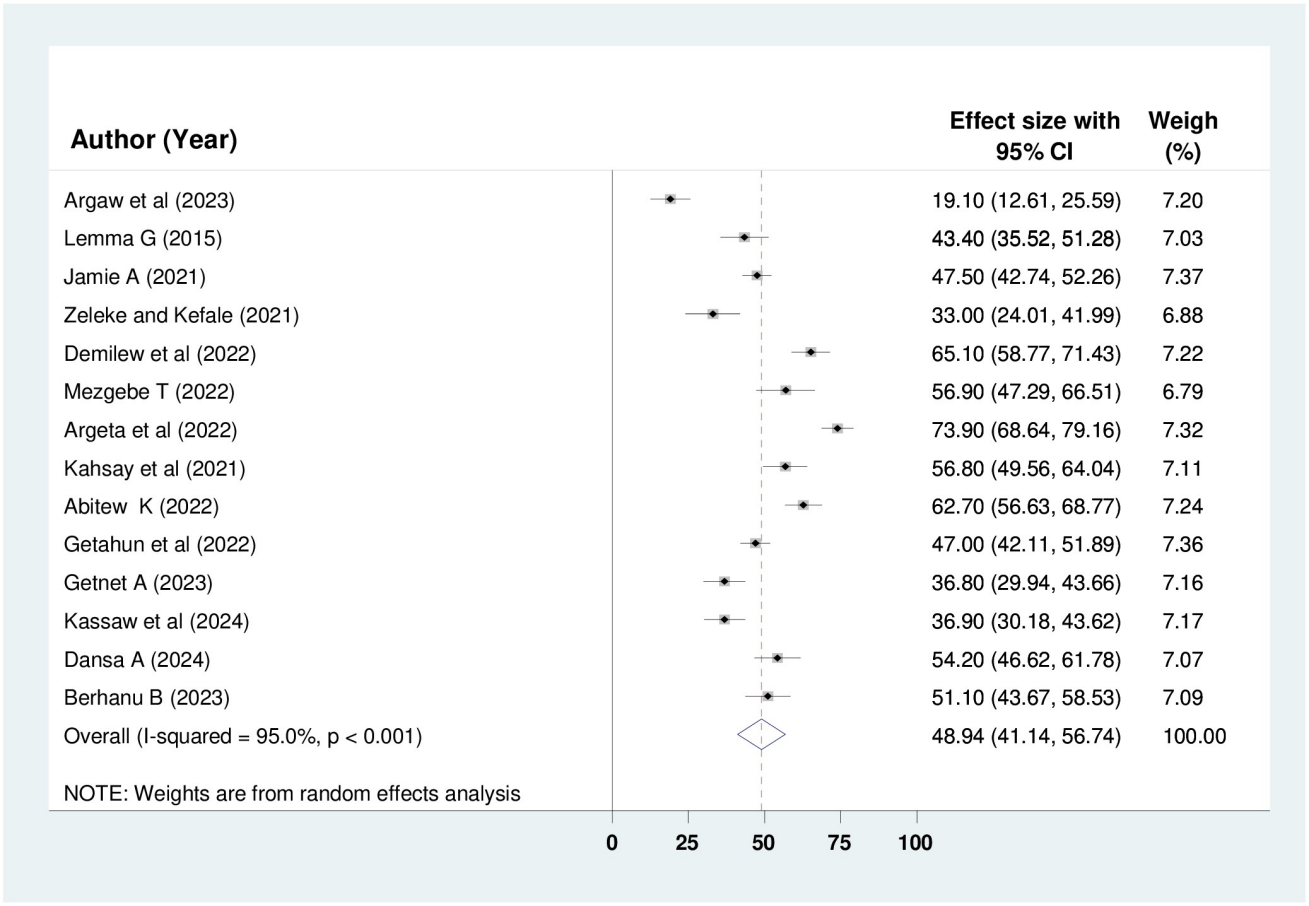
Forest plot showing the pooled prevalence of practice regarding oxygen therapy among HPs in Ethiopia.

Fourteen, 9, and 14 studies involving 2,960, 1,991, and 2,960 study participants were analyzed to determine the pooled prevalence of knowledge, attitude, and practice regarding oxygen therapy among health professionals respectively. By employing the random-effect model, the overall pooled prevalence of good knowledge, positive attitude and good practice among health professionals in Ethiopia was deemed to be 52.13% (95% CI: 43.88%, 60.39%); I^2^ = 95.6%, p < 0.001), 55.08% (95% CI: 50.80%, 59.35%; I^2^ = 73.6%, p < 0.001) and 48.94% (95% CI: 41.14%, 56.74%); I^2^ = 95%, p < 0.001) (Figs 2–4) respectively.

### Subgroup analysis

As mentioned above, Cochran’s Q and I^2^ statistics showed a high level of heterogeneity among the included studies. As a result, to identify the potential source of heterogeneity, the subgroup analysis was computed by considering the study region, study population, sample size, and study quality. The sub-group analysis based on study region, sample size, study population, and study quality revealed that good knowledge was observed at 60.66% in Tigray and Harari regions, 51.92% in studies with a sample size of ≥200, 56.59% among nurses, and 53.18% in studies of high-quality. For attitude, the highest pooled positive attitude was reported in studies from the Amhara region (54.86%) compared to those from Addis Ababa (48.53%). The highest oxygen therapy practice was found in the SNNPR region (60.87%) and among nurses (51.61%) compared to other health professionals (44.21%). Only 38.30% of HPs in Addis Ababa demonstrated good oxygen therapy practice (S4 Table).

### Meta-regression analysis

Apart from conducting subgroup analysis, we employed a meta-regression model to assess the effect of sample size and study year on heterogeneity between the studies. As shown in Table 2, there was no statistically significant prediction of heterogeneity between the effect size and the assessed variables.

**Table 2:** Meta-regression analysis for sample size and year of study as a reason for heterogeneity in the knowledge, attitude, and practice of oxygen therapy among health professionals in Ethiopia.

Outcome variables	Heterogeneity Source	Coefficients (95% CI)	Std. Err.	P-value
Knowledge	Sample size	0.003 (-0.09, 0.10)	0.04	0.92
	Publication year	1.92 (-2.12, 5.97)	1.86	0.32
Attitude	Sample size	0.02 (-0.07, 0.11)	0.04	0.61
	Publication year	-0.54 (-3.32, 2.24)	1.14	0.65
Practice	Sample size	0.04 (-0.08, 0.15)	0.04	0.43
	Publication year	-0.16(-5.19, 6.64)	1.95	0.94

### Sensitivity analysis

Furthermore, sensitivity analyses were computed. The result indicated that no single study unduly influenced the overall estimate of knowledge, attitude, and practice toward oxygen therapy.

### Publication bias

**Fig 5:**
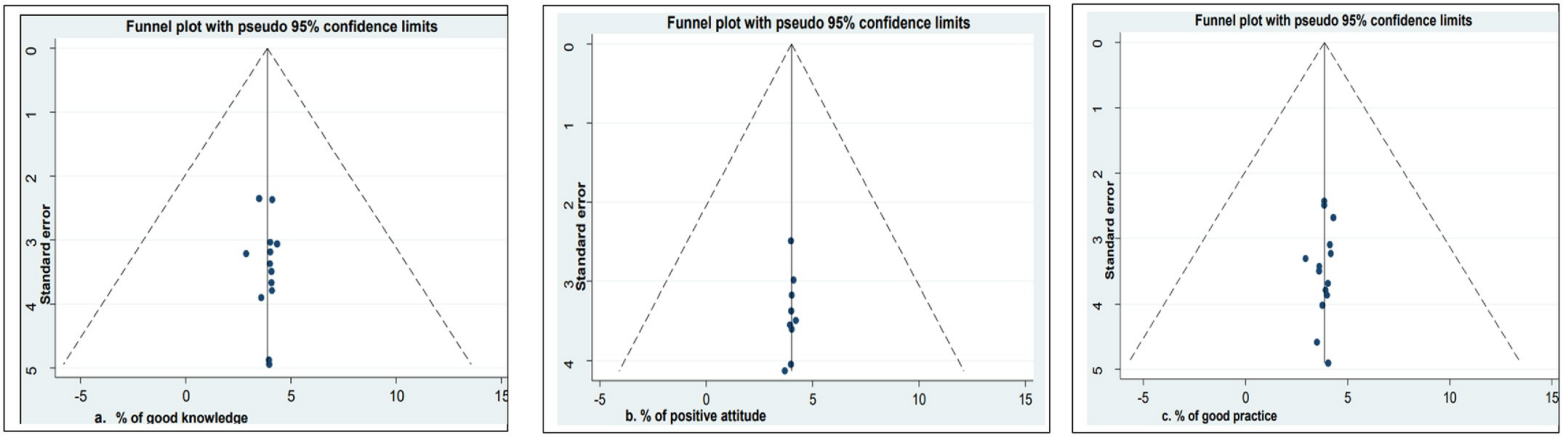
Publication bias assessment: **a)** Funnel plot to check publication bias for the prevalence of knowledge about oxygen therapy, **b)** Funnel plot to assess publication bias for the prevalence of attitude towards oxygen therapy, **c)** Funnel plot to examine publication bias for the prevalence of oxygen therapy practice.

A funnel plot and Egger’s regression test were used to assess publication bias. The funnel plot showed a symmetrical distribution (Fig 5). Again, Egger’s regression test p-values for knowledge, attitude, and practice were 0.73, 0.40, and 0.52 respectively, indicating the absence of publication bias.

### Factors associated with knowledge, attitude and practice about oxygen therapy

Two studies were included to determine the association between the availability of oxygen therapy guidelines and good knowledge about oxygen therapy [39, 55]. The result showed that HPs who had oxygen therapy guidelines in their working department were 6.1 times more likely (AOR: 6.11, 95% CI: 2.45, 15.22) to have good knowledge than those who didn’t have them. No significant variability was observed among the pooled studies (I^2^ = 46.2% and p = 0.173) (S1 Fig). Also, the availability of oxygen therapy guidelines was found to be a significant predictor variable of HPs’ attitude toward oxygen therapy and two cross-sectional studies [53, 55] were assessed to ascertain their associations. Health professionals who had oxygen therapy guidelines were almost 2.2 times more likely (AOR: 2.17, 95% CI: 1.39, 3.39, I^2^ = 0%, p = 0.909) to have a positive attitude towards oxygen therapy compared with those without such guidelines. As shown above, no heterogeneity was observed between the included articles (S2 Fig).

The findings of this meta-analysis revealed the presence of a significant statistical association between HPs’ knowledge and their practice. According to the four pooled studies [24, 35, 53, 56], those HPs who had good knowledge about oxygen therapy were four times (AOR: 4.31, 95% CI: 1.53, 12.11) more likely to have good practice than those who had poor knowledge. Significant heterogeneity was noticed within the studies (I^2^ = 83.4% and p< 0.001). Therefore, the random-effects meta-analysis model was computed (S3 Fig). Moreover, the association between the presence of an adequate oxygen supply and delivery systems and oxygen therapy practice was computed using four studies [35, 39, 49, 53]. Health professionals who had adequate oxygen supply and delivery systems in their health facilities were almost 4 times more likely to have good practices than those who did not have such systems (AOR: 3.12, 95% CI: 1.92, 5.07). No significant heterogeneity was found among the included studies (I^2^ = 37.0%; p = 0.19) (S4 Fig). Additionally, four articles were considered to observe the association between oxygen therapy practice and training [49, 52, 57, 58]. The finding indicated that receiving oxygen therapy training was significantly associated with good practice. Health professionals who received training were 4.1 times more likely to have good practice compared to those who did not receive training (AOR: 4.09, 95% CI: 2.04–8.20; I^2^ = 63.6%, p = 0.061) (S5 Fig).

## Discussion

Oxygen is a frequently prescribed medication in clinical practice and having adequate oxygen therapy knowledge, attitude, and practice are key competencies to safely and effectively provide oxygen therapy to patients in need [39]. Therefore, this systematic review and meta-analysis, which is the first study in Ethiopia as per the knowledge of the researcher, aimed to assess the pooled prevalence of knowledge, attitude, practice, and determinants of oxygen therapy among Ethiopian health professionals. As a result, the pooled prevalence of good knowledge about oxygen therapy among HPs in Ethiopia was 52.13%. Even though there was no comparable meta-analysis study conducted on this topic, this finding was consistent with previous studies conducted in Eritrea [38, 59], India [60], Nigeria [61], and Lebanon [62] revealing that 43.3%, 45%, 48%, 49.5%, and 55.1% of HPs had good knowledge regarding oxygen therapy respectively. Furthermore, another study conducted by Aloushan et al. [2] in Riyadh revealed that the overall good knowledge among health professionals was 55.11%. However, this study finding is lower than the study done in Egypt 76% [37], Sudan 75% [63], Rwanda 73.8% [64], Uganda 76% [65], Turkey 72.99% [66], Pakistan 77.9% [67], Fiji 87% [68] of health professionals had good knowledge regarding oxygen therapy. Besides, the result of this study was higher than studies conducted in Ethiopia [69] where 36.2% of health professionals had good knowledge and in Ghana [34] only 1.10% of them had adequate knowledge. Moreover, this finding is higher than a study done in Rwanda [64] where about 73.8% had a poor level of knowledge regarding oxygen therapy. Variations in oxygen therapy knowledge among HPs across countries can be attributed to differences in education systems, resource allocation, continued professional development, and guidelines availability.

This study found that among Ethiopian health professionals, the pooled prevalence of positive attitudes about oxygen therapy was 55.08%, which was higher than the reported results of two studies conducted in Ghana 37.91% [34] and Sudan 26% [63]. Again, a study by Uwineza [64] in Rwanda showed that 63.1% of health professionals had negative attitudes about oxygen therapy. However, this finding was lower than the results reported by Ghebremichael et al. [38] in Eritrean, Rehman et al. [67] in Pakistan, and Arasi et al. [68] in Fiji, in which 63.3%, 82.9%, and 87.93% health professionals had a positive attitude about oxygen therapy respectively. Differences in the study populations, study setting, study period, and sample size of the reviewed studies may account for this variation.

This review found that among Ethiopian health professionals, the combined prevalence of good practices regarding oxygen therapy was 48.94%. This result was in line with studies from Eritrea [38], Riyadh [2], and Egypt [37], which found that the overall prevalence of good practices among health professionals was 45%, 50.54%, and 58% respectively. However, it was higher than research from Western Ghana (none), Southwest Nigeria (18.8%), Rwanda (32.3%) and Iran (39.1%) of the participants had good practices [34, 61, 64, 70]. In contrast, studies conducted in Sudan found that 73% of respondents had good practices [63], Pakistan found that 92.9% had good practices [67], Fiji found that 84% had good practices [68], Iran found that 74.6% of HPs had good practices [70] and yet another study in Iran found that 74.5% of health professionals had good practices [71]. This disparity could be caused by differences in the availability of oxygen therapy guidelines and protocols, oxygen therapy training, HPs’ knowledge of oxygen therapy, and oxygen delivery and supply systems.

Another objective of this study was to identify factors associated with the KAP of oxygen therapy among health professionals in Ethiopia. The findings of this study revealed that both good knowledge and positive attitude were affected by the availability of oxygen therapy guidelines. Moreover, the presence of an adequate oxygen supply and delivery system, training, and good knowledge were significantly associated with good practice.

The odds of good knowledge were 6.11 times higher among health professionals who had oxygen therapy guidelines at working units compared to those who didn’t have such guidelines. This finding was supported by studies done in Riyadh [2] and Egypt [37]. Evidence backs up this notion by indicating that, to provide oxygen therapy safely and effectively, it is essential to follow the WHO and British Thoracic Society oxygen therapy [14, 61]. A possible explanation could be that guidelines serve as a valuable educational tool [72]. When HPs have access to these guidelines, it facilitates the development of their knowledge about oxygen therapy. A wide range of topics are usually covered by these guidelines such as indications for oxygen therapy, administration methods, dosages, monitoring techniques, and potential side effects. As a result, they can familiarize themselves with the latest recommendations and explore various scenarios where oxygen therapy might be necessary.

When compared to health professionals without oxygen therapy guidelines, those who had such guidelines in their working units were almost 2.2 times more likely to have a positive attitude toward oxygen therapy. This finding was similar to a previous study done by Ghebremichael *et al*. [38] in Eritrea that revealed a lack of oxygen therapy guidelines attributed to a negative attitude. The possible explanation could be that when healthcare workers have access to oxygen therapy guidelines, they feel more confident and satisfied in their practice because it helps them reduce any anxiety or uncertainty that may arise when managing patients requiring oxygen therapy and enables them to make informed decisions based on evidence-based recommendations. Moreover, guidelines provide a framework for healthcare professionals to communicate and align their practices with colleagues, interdisciplinary teams, and supervisory bodies. This assists them to feel more confident and satisfied with their clinical judgment. All this can have a positive impact on their attitude towards oxygen therapy.

When comparing HPs with their counterparts, those with good knowledge of oxygen therapy had four times higher odds of having good practice. Studies by Adeniyi et al. [61] and Piryani et al. [73] found a significant correlation between oxygen therapy knowledge and practice; inferring that those health professionals with good knowledge perform better in the field of practice. This may be because healthcare workers with good oxygen therapy knowledge are more likely to be on top of things. They can accurately assess a patient’s oxygen needs by evaluating vital signs, symptoms, and oxygen saturation levels; understand the different delivery methods and select the most suitable one based on the patient’s needs; closely monitor patients receiving oxygen therapy; and implement measures to prevent oxygen toxicity. Additionally, knowledgeable healthcare professionals are more likely to adhere to best practices and evidence-based guidelines, they understand the importance of proper documentation and appropriate titration of oxygen levels based on patient response to therapy, all of which contribute to better oxygen therapy practices.

Furthermore, HPs who had adequate oxygen supply and delivery systems in their health facilities were 3.1 times more likely to have good practices than those who did not have such systems. In agreement with this finding, Mayhob [37] reported that the most frequent hurdles influencing health professionals’ practice of administering oxygen were insufficient supplies, a lack of periodic maintenance, and the existence of malfunctioning equipment in health facilities. Again, evidence revealed that most patients in need of oxygen did not receive it because of a subpar supply and delivery system [74, 75] and concluded that most facilities in sub-Saharan Africa are ill-equipped with supplies and involving stakeholders in the issue is essential to finding a solution [76]. One explanation for this could be that a consistent oxygen supply system and adequate oxygen availability are paramount for HPs when administering oxygen. Access to oxygen in a timely and uninterrupted manner enhances overall patient care, enables rapid response to emergencies, supports medical procedures, manages critical conditions, and facilitates resource allocation during crises. Additionally, a continuous oxygen therapy supply allows HPs to treat patients as needed. Some patients may require round-the-clock oxygen therapy to manage chronic conditions. In such cases, a steady and uninterrupted supply of oxygen is essential to maintain optimal oxygen levels and support the patient’s respiratory function. Collectively, these may affect the health professionals’ oxygen therapy practices.

The findings of this study indicate a significant association between receiving oxygen therapy training and demonstrating good practice. Health professionals who received training were 4.1 times more likely to have good practice compared to those who did not receive such training. This finding aligns with earlier studies [26, 29, 77], which found that educational interventions can enhance health professionals’ skills in oxygen therapy.

This systematic review and meta-analysis has several limitations and strengths. First, heterogeneity between studies was high. Nonetheless, a series of subgroup, meta-regression, and sensitivity analyses were conducted to address this problem. Second, articles not published in English were excluded, which may result in the exclusion of some studies. Finally, all included studies were cross-sectional, which might share the nature of cross-sectional study design limitations. Despite these limitations, to our knowledge, this is the first systematic review and meta-analysis on KAP of oxygen therapy among healthcare professionals in Ethiopia.

## Conclusion and recommendations

The national pooled prevalence of good knowledge, positive attitude, and good practice among healthcare professionals was low. Both good knowledge and positive attitudes were affected by the availability of oxygen therapy guidelines. Moreover, the presence of an adequate oxygen supply and delivery system, good knowledge, and training are statistically associated with good practice. Therefore, thorough monitoring, supervision, and evaluation of their oxygen therapy is highly recommended for all stakeholders. Once more, we strongly advise that the identified factors be improved by organizing training sessions, making oxygen therapy guidelines and protocols available, and maintaining adequate oxygen supply and delivery systems in the health facilities.

## Supporting information

S1 Checklist PRISMA 2020 checklist. (DOCX)

S2 Checklist AMSTAR checklist. (PDF)

S1 Table Searching strategies. (DOCX)

S2 Table JBI Critical appraisal checklist for eligible studies. (DOCX)

S3 Table List of excluded full texts with reasons. (DOCX)

S4 Table Subgroup analysis. (DOCX)

S1 Fig The association between availability of oxygen therapy guidelines and good knowledge. (TIF)

S2 Fig The association between availability of oxygen therapy guidelines and positive attitude. (TIF)

S3 Fig The association between good knowledge and good practice. (TIF)

S4 Fig The association between adequate oxygen supply and delivery system and good practice. (TIF)

S5 Fig The association between training and good practice. (TIF)

S1 Data (XLSX)
