# Supplementary material for: Knowledge, attitude, practice and associated factors of oxygen therapy among health professionals in Ethiopia: A systematic review and meta-analysis
Source: PLoS One. 2024 Sep 6;19(9):e0309823. doi: 10.1371/journal.pone.0309823 (PMC11379292; doi:10.1371/journal.pone.0309823)
Supplement: S1 Table — (DOCX) [file pone.0309823.s003.docx]

**S1 Table:** Searching strategies for some databases to assess the pooled knowledge, attitude, practice, and associated factors of oxygen therapy among health professionals in Ethiopia

| **Databases** | **Searching terms** | **Hits** |
| --- | --- | --- |
| PubMed | ("Knowledge"[All Fields] OR "Attitude"[All Fields] OR "Practice"[All Fields]) AND ("Oxygen Therapy"[All Fields] OR "Oxygen"[All Fields] OR "Therapy"[All Fields] OR “Oxygen administration”[All Fields] AND ("Health Professionals"[All Fields] OR "Healthcare Workers"[All Fields] OR "Medical Staff"[All Fields]) AND "Ethiopia"[All Fields] | 146 |
| Google scholar | "Knowledge" AND "Attitude" AND "Practice" AND "Associated Factors" AND "Oxygen Therapy" AND "Health Professionals" AND "Ethiopia" | 57 |
| ScienceDirect | Knowledge, attitude, practice and associated factors of oxygen therapy among health professionals in Ethiopia | 11 |
| Scopus | Knowledge, attitude, practice and associated factors of oxygen therapy among health professionals in Ethiopia | 8 |
| Web of Science | Knowledge, attitude, practice and associated factors of oxygen therapy among health professionals in Ethiopia | 13 |
| African Journal of Online | Knowledge, attitude, practice and associated factors of oxygen therapy among health professionals in Ethiopia | 19 |
| HINARI | Knowledge, attitude, practice and associated factors of oxygen therapy among health professionals in Ethiopia | 85 |
| Gray literature (for unpublished studies) | Knowledge, attitude, practice and associated factors of oxygen therapy among health professionals in Ethiopia | 4 |
| **Total searched articles** | | 343 |
| **Total included articles** | | 15 |
