# Supplementary material for: Knowledge, attitude, practice and associated factors of oxygen therapy among health professionals in Ethiopia: A systematic review and meta-analysis
Source: PLoS One. 2024 Sep 6;19(9):e0309823. doi: 10.1371/journal.pone.0309823 (PMC11379292; doi:10.1371/journal.pone.0309823)
Supplement: S2 Table — (DOCX) [file pone.0309823.s004.docx]

**S2 Table:** JBI Critical appraisal checklist for eligible studies

| **Studies** | **Q1** | **Q2** | **Q3** | **Q4** | **Q5** | **Q6** | **Q7** | **Q8** | **Q9** | **Overall score** | **Covert to 100%** | **Quality** |
| --- | --- | --- | --- | --- | --- | --- | --- | --- | --- | --- | --- | --- |
| Argaw et al (2023) | Y | Y | N | Y | Y | Y | Y | Y | Y | 8 | 88.9 | High |
| Lemma G (2015) | Y | N | N | Y | Y | Y | Y | N | Y | 7 | 77.8 | Modest |
| Jamie A (2021) | Y | Y | Y | N | Y | Y | Y | Y | Y | 8 | 88.9 | High |
| Zeleke and Kefale (2021) | Y | Y | N | Y | Y | Y | Y | Y | Y | 8 | 88.9 | High |
| Demilew et al (2022) | Y | Y | Y | Y | Y | Y | Y | Y | Y | 9 | 100 | High |
| Abitew K (2022) | Y | Y | Y | N | Y | Y | Y | Y | Y | 8 | 88.9 | High |
| Mezgebe T (2022) | Y | N | N | Y | Y | Y | Y | Y | Y | 7 | 77.8 | Modest |
| Bizuneh et al (2022) | Y | Y | Y | Y | Y | Y | Y | Y | Y | 9 | 100 | High |
| Getahun et al (2022) | Y | Y | Y | Y | Y | Y | Y | Y | Y | 9 | 100 | High |
| Argeta et al (2022) | N | N | Y | Y | Y | Y | Y | Y | Y | 7 | 77.8 | Modest |
| Kahsay et al (2021) | Y | Y | N | Y | Y | Y | Y | Y | Y | 8 | 88.9 | High |
| Getnet A (2023) | Y | Y | N | Y | Y | Y | Y | Y | Y | 8 | 88.9 | High |
| Kassaw et al (2024) | Y | Y | N | Y | Y | Y | Y | Y | Y | 8 | 88.9 | High |
| Dansa et al (2024) | Y | Y | N | Y | Y | Y | Y | Y | Y | 8 | 88.9 | High |
| Bezawit B (2023) | Y | Y | N | Y | Y | Y | Y | Y | Y | 8 | 88.9 | High |

**Key:** Y, yes = 1; N, no = 0; JBI = Joanna Briggs Institute; overall score is calculated by counting the number of Y’s in each row.

**Notes:**

Q1 - Was the sample frame appropriate to address the target population?

Q2 - Were study participants sampled in an appropriate way?

Q3 - Was the sample size adequate?

Q4 - Were the study subjects and the setting described in detail?

Q5 - Was the data analysis conducted with sufficient coverage of the identified sample?

Q6 - Were valid methods used for the identification of the condition?

Q7 - Was the condition measured in a standard, reliable way for all participants?

Q8 - Was there appropriate statistical analysis?

Q9 - Was the response rate adequate, and if not, was the low response rate managed appropriately?
