# Supplementary material for: Knowledge, attitude, practice and associated factors of oxygen therapy among health professionals in Ethiopia: A systematic review and meta-analysis
Source: PLoS One. 2024 Sep 6;19(9):e0309823. doi: 10.1371/journal.pone.0309823 (PMC11379292; doi:10.1371/journal.pone.0309823)
Supplement: S3 Table — (DOCX) [file pone.0309823.s005.docx]

**S3 Table: List of excluded full texts with reasons for exclusion**

| **Author, year** | **Reason for exclusion** |
| --- | --- |
| Sultan M, 2023 [1] | The outcome of interest is not clearly reported |
| Nigatu M et al, 2022 [2] | Different outcomes |
| Lema G, 2017 [3] | Duplicate publication |
| Uwineza D, 2017 [4] | Conducted out of Ethiopia |

**References of excluded studies**1. Sultan M. Knowledge, Attitude and Practice of Physicians and Nurses Working in Addis Ababa on Oxygen therapy. Pan African Journal of Emergency Medicine and Critical Care. 2023;1(2).

2. Nigatu M, Debebe F, Tuli W. Assessment of Knowledge, Practice, and Associated Factors Towards Airway and Breathing Management Among Nurses Working in the Emergency Departments of Selected Public Hospitals in Addis Ababa, Ethiopia: A Cross-Sectional Study. Open Access Emergency Medicine. 2022:235-47.

3. Lema G, Beza L. Knowledge, attitude and practice study of oxygen therapy among emergency department nurses in Addis Ababa, Ethiopia. Prehospital and Disaster Medicine. 2017;32(S1):S149-S.

4. Uwineza Didi V. Knowledge, attitudes and practice among nurses toward oxygen administration to the critically ill patients at UTHK: University of Rwanda; 2017.
