# Supplementary material for: Knowledge, attitude, practice and associated factors of oxygen therapy among health professionals in Ethiopia: A systematic review and meta-analysis
Source: PLoS One. 2024 Sep 6;19(9):e0309823. doi: 10.1371/journal.pone.0309823 (PMC11379292; doi:10.1371/journal.pone.0309823)
Supplement: S4 Table — (DOCX) [file pone.0309823.s006.docx]

**S4 Table:** Subgroup pooled prevalence of oxygen therapy knowledge, attitude and practice among health professionals in Ethiopia.

| **Outcome variables** | **Variables** | **Subgroups** | **Number of studies** | **Prevalence in % (95% CI)** | **I^2^, P-value** |
| --- | --- | --- | --- | --- | --- |
| Knowledge | Region | Tigray and Harari | 2 | 60.66 (56.76, 64.56) | 0%, 0.567 |
|  |  | SNNPR | 3 | 55.13 (50.95, 59.31) | 0%, 0.882 |
|  |  | Amhara | 5 | 54.31 (38.19, 70.43) | 97%, <0.001 |
|  |  | Addis Ababa | 4 | 43.42 (22.35, 64.48) | 97.2%, <0.001 |
|  | Sample size | ≥200 | 5 | 51.92 (40.73, 63.12) | 95.2%, <0.001 |
|  |  | <200 | 9 | 52.24 (39.69, 64.78) | 96.2%, <0.001 |
|  | Population | Nurses | 9 | 56.59 (47.65, 65.52) | 94.6%, <0.001 |
|  |  | Others | 5 | 44.03 (27.67, 60.39) | 96.1%, <0.001 |
|  | Study quality | High | 11 | 53.18 (43.19, 63.17) | 96.4%, <0.001 |
|  |  | Modest | 3 | 48.25 (35.73, 60.77) | 87.6%, <0.001 |
| Attitude | Region | Amhara | 4 | 54.86 (51.86, 57.87) | 0%, 0.939 |
|  |  | Addis Ababa | 3 | 48.53 (40.91, 56.15) | 66.4%, 0.051 |
|  | Sample size | ≥200 | 4 | 56.20 (53.05, 59.35) | 15.1%, 0.317 |
|  |  | <200 | 5 | 53.82 (45.41, 62.23) | 84.7%, < 0.001 |
|  | Population | Nurses | 5 | 58.58 (53.91, 63.24) | 65.8%, 0.020 |
|  |  | Others | 4 | 50.18 (44.17, 56.20) | 62.5%, 0.046 |
|  | Study quality | High | 7 | 54.41 (49.21, 59.60) | 77.3%, 0.001 |
|  |  | Modest | 2 | 57.55 (50.26, 64.83) | 55.1%, 0.136 |
| Practice | Region | SNNPR | 3 | 60.87 (45.42, 76.33) | 92.6%, < 0.001 |
|  |  | Amhara | 5 | 49.13 (37.36, 60.90) | 94.1%, < 0.001 |
|  |  | Tigray and Harari | 2 | 51.73 (42.66, 60.81) | 77.4%, 0.035 |
|  |  | Addis Ababa | 4 | 38.30 (23.53, 53.08) | 94.2%, < 0.001 |
|  | Sample size | ≥200 | 5 | 59.17 (48.36, 69.98) | 95%, < 0.001 |
|  |  | <200 | 9 | 43.03 (34.29, 51.76) | 91.8%, <0.001 |
|  | Population | Nurses | 9 | 51.61 (43.43, 59.79) | 93.5%, < 0.001 |
|  |  | Others | 5 | 44.21 (27.24, 61.17) | 96.4%, < 0.001 |
|  | Study quality | High | 11 | 46.44 (38.54, 54.34) | 94.0%, < 0.001 |
|  |  | Modest | 3 | 58.24 (38.63, 77.85) | 95.2%, < 0.001 |

**Key:** SNNPR = Southern Nations, Nationalities and Peoples Region; Others = Anesthetists, IESO, Midwives, Nurses and Physicians; IESO = Integrated Emergency Surgical Officers
