# Supplementary figures and images for: Knowledge, attitude, practice and associated factors of oxygen therapy among health professionals in Ethiopia: A systematic review and meta-analysis
Source: PLoS One. 2024 Sep 6;19(9):e0309823. doi: 10.1371/journal.pone.0309823 (PMC11379292; doi:10.1371/journal.pone.0309823)

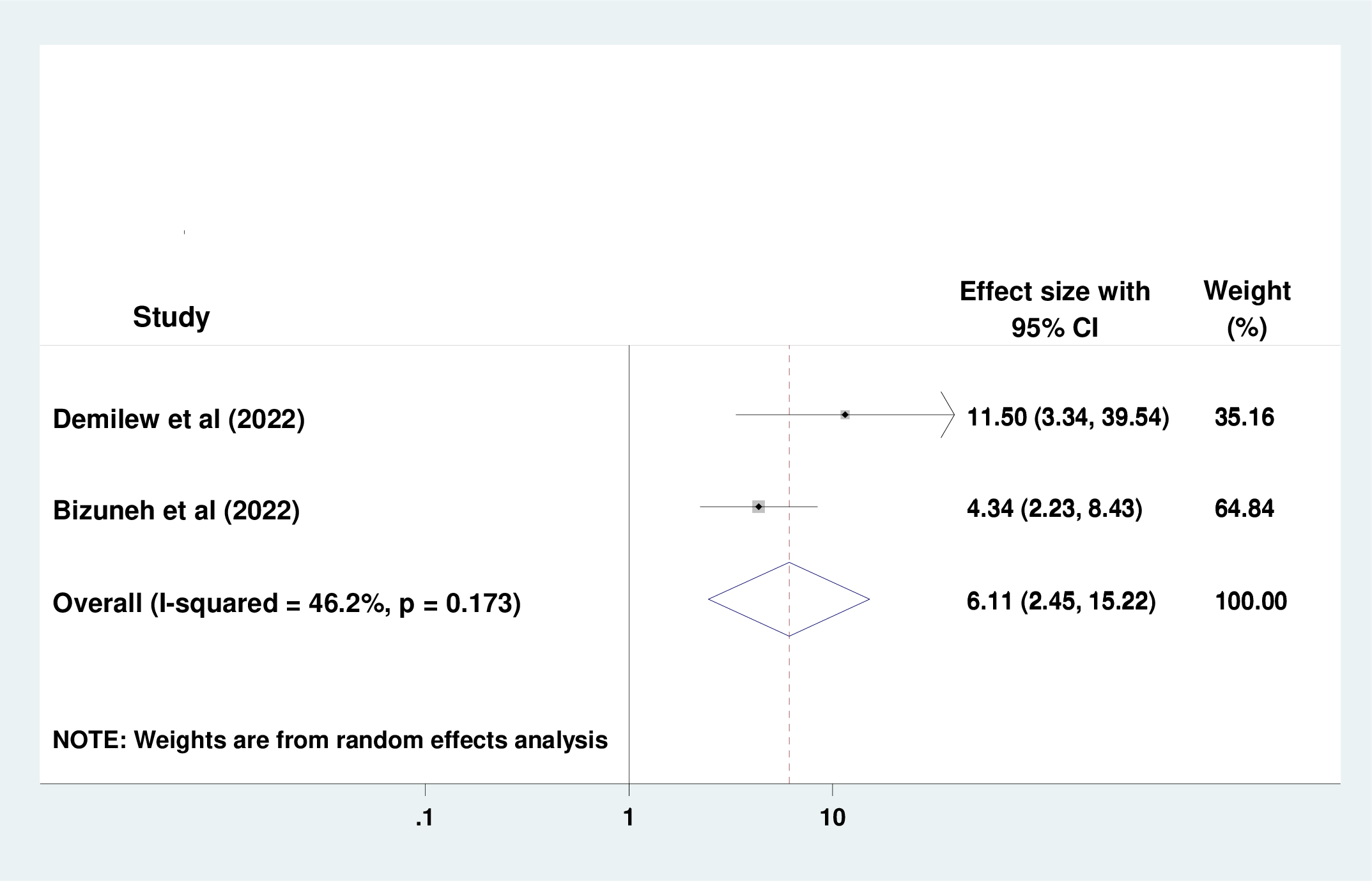

Supplement: S1 Fig — (TIF) [file pone.0309823.s007.tif]

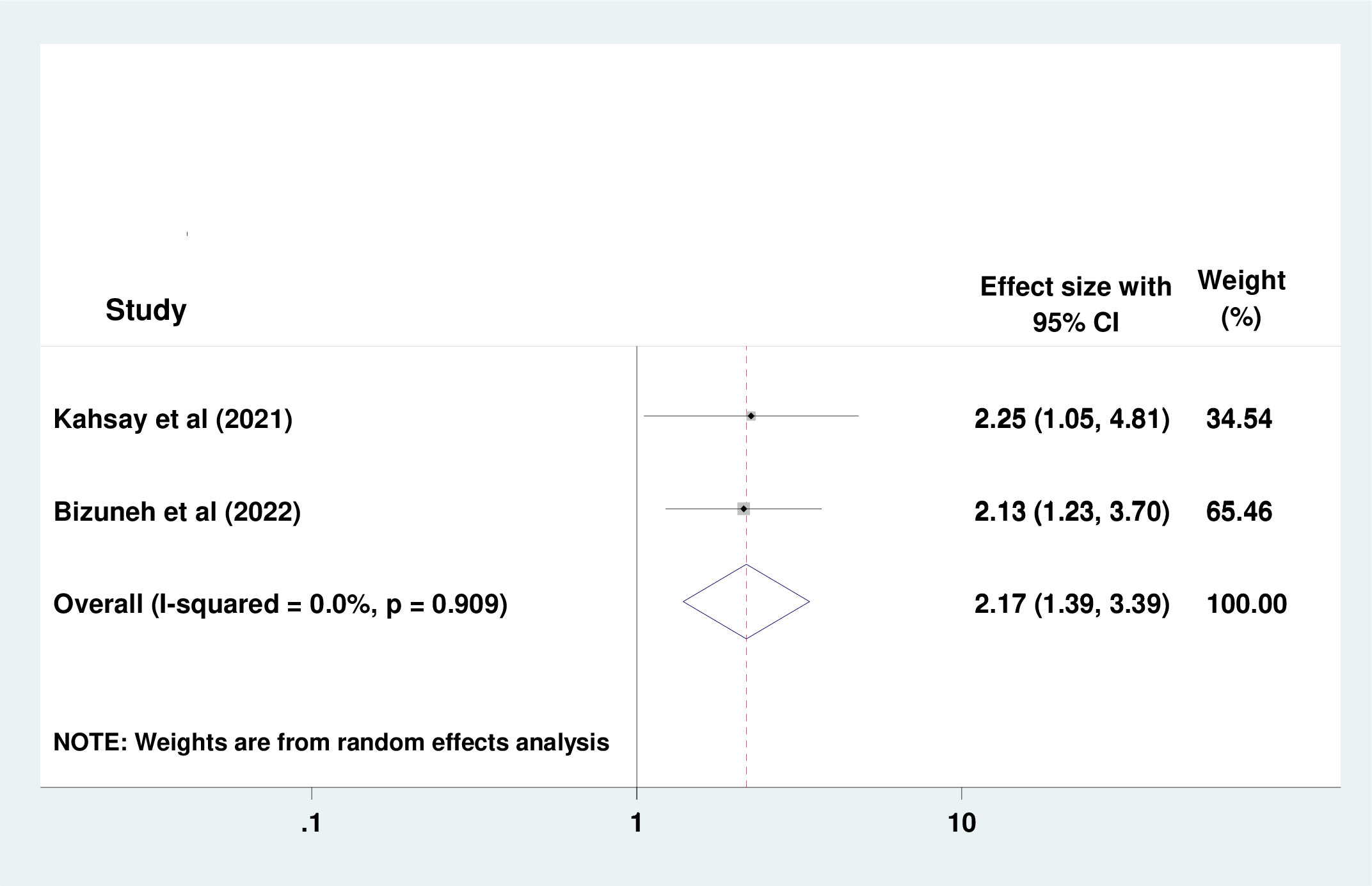

Supplement: S2 Fig — (TIF) [file pone.0309823.s008.tif]

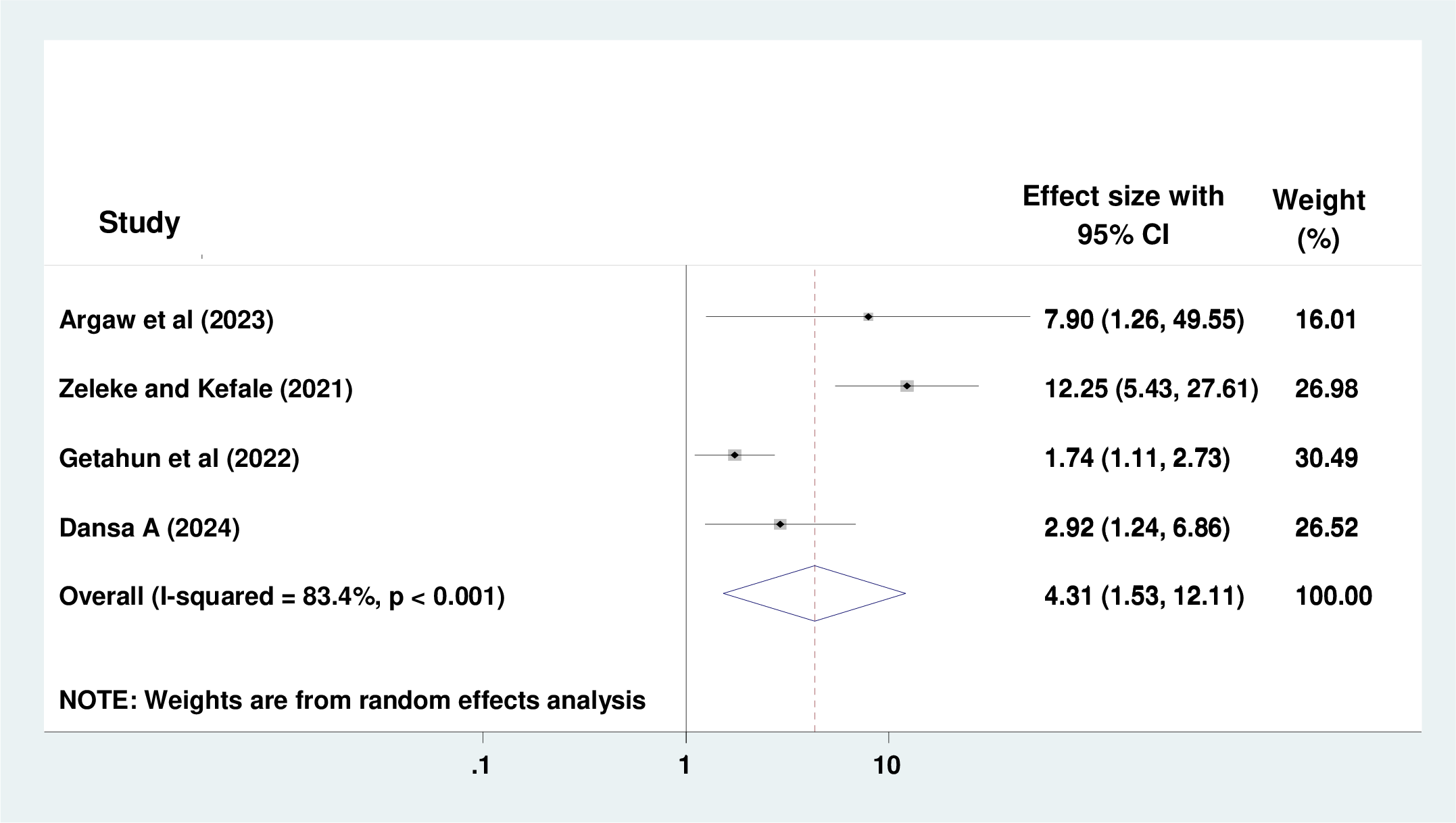

Supplement: S3 Fig — (TIF) [file pone.0309823.s009.tif]

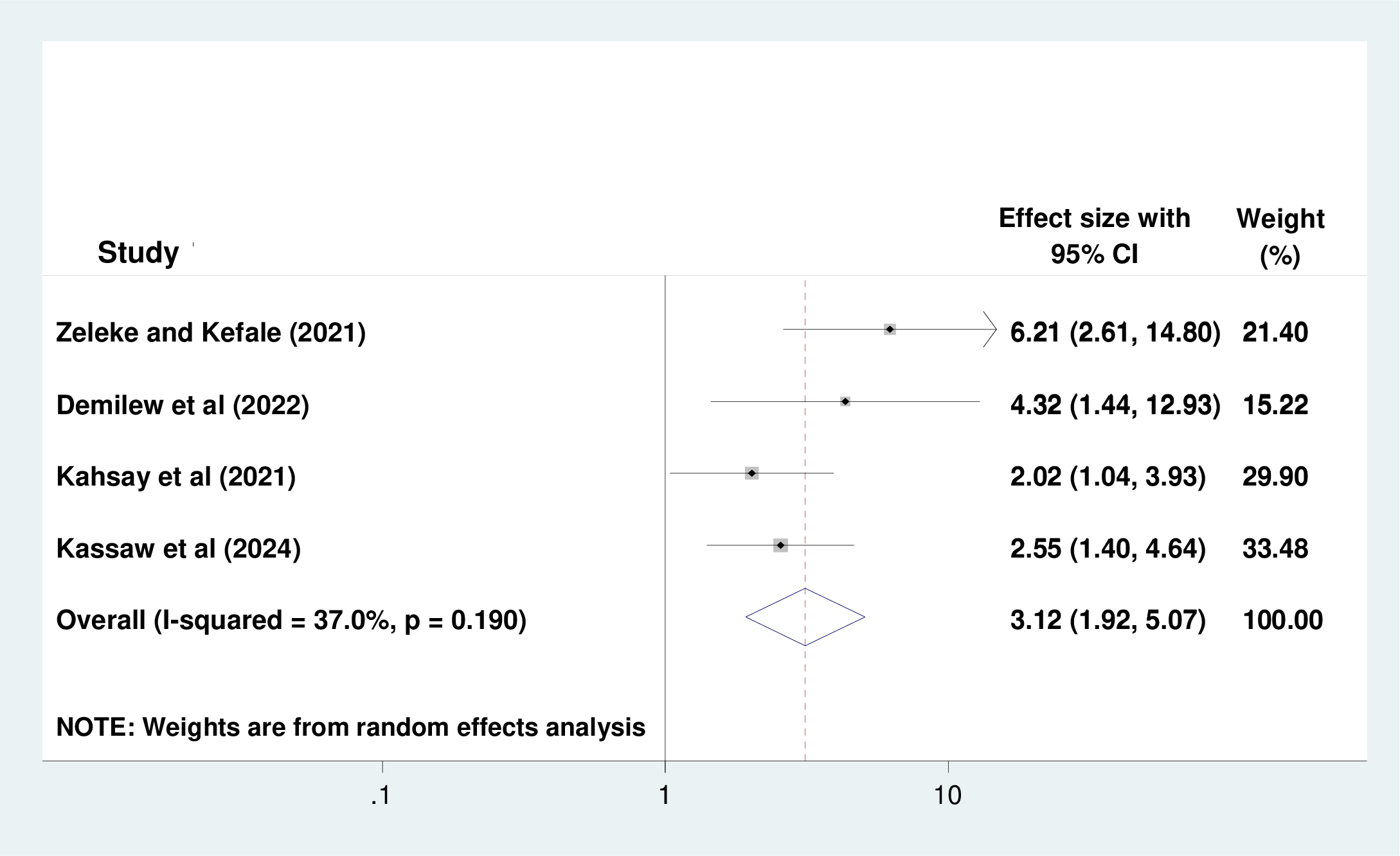

Supplement: S4 Fig — (TIF) [file pone.0309823.s010.tif]

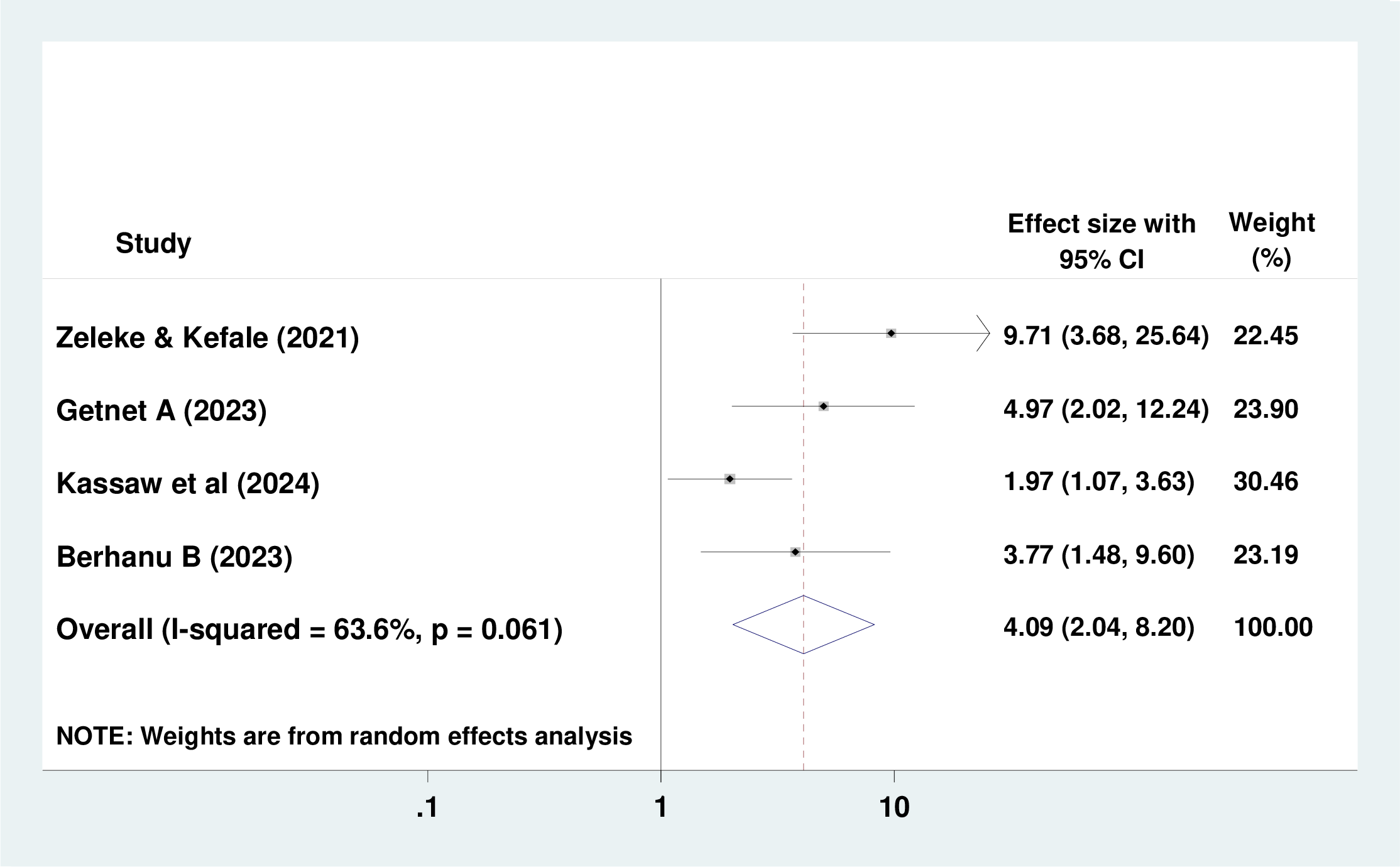

Supplement: S5 Fig — (TIF) [file pone.0309823.s011.tif]
